# Conjunctivitis

**Published:** 1907-03

**Authors:** 


### CONJUNCTIVITIS.

The most common causes of conjunctivitis in children, accoring to the Practitioner, are the Koch-Weeks bacillus, the Morax-Axenfeld bacillus, the gonoccoccus of Neisser, and the pneumococcus of Fraenkel. In rare cases the streptococcus, the staphylococcus and the bacillus pneumoniæ may bze producing factors.

The treatment should first consist of warning the child's parents of the necessity of isolating the child on account of the contagious nature of the disease and at the same time of insisting that all towels, cloths and sponges used by the patient should be kept exclusively for the patient and afterward boiled or burned.

Secondly, local treatment must be applied to the eye, from which the discharge should be carefully washed away four or five times a day. This procedure can be carried out, according to the Practitioner, by soaking a pledget of absorbent cotton-wool in a proper antiseptic solution and by gently squeezing so as to produce a continuous stream and allowing it to drop into the inner canthus. The patient should be instructed to lie down and the eye should be gently opened by the person bathing it.

The following lotions are recommended for this purpose:

| | | | | |
|---|---|---|---|---|
| ℞ | Hydrarg. Perchloridi | gr. 1-16 | | 004 |
| | Aquæ dest. | f℥i | 30 | |
| M. | Ft. collyrium. S.: Apply locally. Or: | | | |
| ℞. | Acidi borici | gr. x | | 65 |
| | Zinci sulphatis | gr. ii | | 12 |
| | Aquæ dest. q. s. ad. | f℥i | 30 | |
| M. | Ft. collyrium. Sig.: Apply locally twice or three times a day. Or: | | | |
| ℞. | Sodii biboratis | gr. vi | | 40 |
| | Acidi salicylici | gr. ii | | 12 |
| | Aquæ dest. .. s. ad | f℥i | 30 | |
| M. | Ff. collyrium. Sig.: Apply locally | | | |

In some cases a weak solution of silver nitrate is very useful. It is recommended in the following strength solution:

| | | |
|---|---|---|
| ℞. Argenti niratis | gr. ii | 12 |
| Aquæ dest | f℥i | 30 |

M. Ft. collyrium. Sig.: One or two drops to be dropped into the inner canthus once or twice a day.

It is also recommended that a little spermaceti ointment be painted along the margins of the lids with a clean camel's hair brush after all the discharge has been bathed away in order to prevent the lids from sticking together during sleep. The eyes should not be bandaged, as the discharge should be allowed to escape freely. Corneal complications seldom arise, although one or more phylctenulæ may occur.—Journal A. M. A., January 19, 1907.

